# Tick-Borne Encephalitis Virus: An Emerging Ancient Zoonosis?

**DOI:** 10.3390/v12020247

**Published:** 2020-02-23

**Authors:** Andrei A. Deviatkin, Ivan S. Kholodilov, Yulia A. Vakulenko, Galina G. Karganova, Alexander N. Lukashev

**Affiliations:** 1 Laboratory of Molecular Biology and Biochemistry, Institute of Molecular Medicine, Sechenov First Moscow State Medical University, 119048 Moscow, Russia; alexander_lukashev@hotmail.com; 2 Laboratory of Postgenomic Technologies, Izmerov Research Institute of Occupational Health, 105275 Moscow, Russia; 3 Laboratory of Biology of Arboviruses, Chumakov Institute of Poliomyelitis and Viral Encephalitides (FSBSI “Chumakov FSC R&D IBP RAS), 108819 Moscow, Russia; ivan-kholodilov@bk.ru (I.S.K.); karganova@bk.ru (G.G.K.); 4 Martsinovsky Institute of Medical Parasitology, Tropical and Vector Borne Diseases, Sechenov First Moscow State Medical University, 119435 Moscow, Russia; vjulia94@gmail.com; 5 Department of Virology, Faculty of Biology, Lomonosov Moscow State University, 119234 Moscow, Russia; 6 Department of Organization and Technology of Immunobiological Preparations, Institute for Translational Medicine and Biotechnology, Sechenov First Moscow State Medical University, 119991 Moscow, Russia

**Keywords:** TBEV, flavivirus, Bayesian phylogeny, temporal signal, population growth

## Abstract

Tick-borne encephalitis (TBE) is one of the most important viral zoonosis transmitted by the bite of infected ticks. In this study, all tick-borne encephalitis virus (TBEV) E gene sequences available in GenBank as of June 2019 with known date of isolation (*n* = 551) were analyzed. Simulation studies showed that a sample bias could significantly affect earlier studies, because small TBEV datasets (*n* = 50) produced non-overlapping intervals for evolutionary rate estimates. An apparent lack of a temporal signal in TBEV, in general, was found, precluding molecular clock analysis of all TBEV subtypes in one dataset. Within all subtypes and most of the smaller groups in these subtypes, there was evidence of many medium- and long-distance virus transfers. These multiple random events may play a key role in the virus spreading. For some groups, virus diversity within one territory was similar to diversity over the whole geographic range. This is best exemplified by the virus diversity observed in Switzerland or Czech Republic. These two countries yielded most of the known European subtype Eu3 subgroup sequences, and the diversity of viruses found within each of these small countries is comparable to that of the whole Eu3 subgroup, which is prevalent all over Central and Eastern Europe. Most of the deep tree nodes within all three established TBEV subtypes dated less than 300 years back. This could be explained by the recent emergence of most of the known TBEV diversity. Results of bioinformatics analysis presented here, together with multiple field findings, suggest that TBEV may be regarded as an emerging disease.

## 1. Introduction

Tick-borne encephalitis (TBE) is the most prevalent viral tick-borne zoonosis in Europe and Northern Asia. Over the last 10 years, the annual number of cases has been about 2000–3000 per year in the European Union and European Economic Area and 1500–2000 in Russia [1,2]. TBE is prevalent over a so-called “TBE belt,” which extends across Eurasia from Central Europe to the Pacific [3]. About 30% of severe infection cases result in neurological complications, and in Russia alone, there are 20–100 lethal outcomes per year. The number of mild and subclinical cases may be much higher than reported, because several studies found relatively high TBE virus (TBEV) seroprevalence rates among the general population in endemic areas [4,5].

The disease is caused by the TBEV, a member of the genus *Flavivirus* in the family Flaviviridae. The virus is enveloped and has a single-stranded RNA genome of about 11,100 bases, which encodes for a large open reading frame. The polyprotein is processed into structural proteins *n* (core or nucleocapsid), E (envelope) and prM/M, and non-structural proteins termed NS1–NS5. The major antigenic determinants lie within the E protein, and the corresponding genome region is the most commonly used for phylogenetic studies.

About 20 years ago, the TBEV was divided into three main subtypes based on the phylogenetic analysis: European, Siberian, and Far-Eastern [6]. Louping ill virus, a tick-borne ovine flavivirus endemic to many areas in Europe, from the British Isles to the Mediterranean, is a sister clade to the European TBEV subtype [7]. The geographic distribution of subtypes mostly corresponds to the nominal region. However, there are some exceptions. The European subtype has been found in the territory of South Korea [8], Altai, and Irkutsk Region. The Siberian variant circulates in Scandinavia [9], the Baltic [10], Sakhalin [11], Bosnia [12], and Central Asia [13,14]. The Far-Eastern subtype has been detected in Southern Siberia, in the Urals, in the Baltic [10], and in Moldova [15]. All three main subtypes of the TBEV have been found on the Crimean Peninsula [16]. Two divergent TBEV strains 178-179 and 886-84 have been described recently in the Irkutsk area, Transbaikalia, and Mongolia [17]. Although these strains form a common clade with the Far-Eastern subtype, some authors consider them as two separate subtypes of TBEV [18,19]. In 2018, another TBEV subtype was described in marmots in the Tibetan Highlands [18]. In 2016, a new lineage of the European subtype of TBEV was found in the Netherlands, a country previously considered as free of TBE [20]. The Dutch variant differs from all other European subtype isolates by at least 8% nucleotide distance.

In nature, TBEV circulates within stable natural foci, which are formed by a tick vector and several mammalian hosts [21]. Typical hosts for larvae are small mammals, such as small rodents and insectivores [22]. Birds and large mammals in turn may serve as hosts for the adult ticks. The major virus vectors are *Ixodes ricinus* and *I. persulcatus* ticks. These ticks are the main sources of human infection for European (*I. ricinus*), Siberian, and Far-Eastern subtypes (*I. persulcatus*). Other ticks have been implicated as TBEV vectors in Siberia and the Far East [23,24]; however, their role in global-scale TBEV ecology is not clear. There have been several hypotheses regarding the evolutionary history of the TBEV. The most common one includes emergence from a common ancestor with the Omsk hemorrhagic fever virus in Central Eurasia about 3000 years ago and then eastward spread of the Far-Eastern subtype, spread of the Siberian subtype to the East and West and westward spread of the European subtype [25].

After the rapid increase in the incidence of TBE in the 1990s, starting in 1999, there was a steady decline in this indicator in Russia [2,26,27], whereas an increase in TBE occurrence was observed in the European Economic Area in 2000s [27]. Then, in the 2010s, TBE incidence rate stabilized in Europe [1]. The reasons behind such changes are not known and may include decreasing activity of natural foci as a part of the well-known cyclic fluctuations in TBE incidence, increasing vaccination coverage, and improving safety habits among population in endemic regions. However, in certain areas, the incidence of TBE has been increasing, and it appears as an emerging disease. Increase of average annual temperature over the last few decades resulted in the northward spread of the forest that is replacing tundra in the Kola Peninsula and the Arkhangelsk region in Russia. This was supposed to be followed by the introduction of mammal hosts and tick vectors of TBEV [28] and led to emergence of TBE in new regions at the northern border of the area [28,29]. In 1980–2000, TBE emerged in the Arkhangelsk region, and the incidence increased from almost zero to about 2 per 100,000 population, a typical rate in a moderately endemic region. Between 2000 and 2009, there was a further five-fold increase of TBE incidence in the Arkhangelsk region [29]. The emergence of morbidity in the previously TBE-free areas corresponded to the northward expansion of the *I. persulcatus* area and posed a challenge for healthcare, because at that time, it was not known how the incidence dynamics would develop [29]. In Karelia, there has been an overall increase of prevalence of ticks and invasion of *I. persulcatus* into areas that were previously endemic for *I. ricinus* [30]. This was paralleled by an approximately two- to four-fold increase in TBE incidence between 1997 and 2003–2008. Similarly, in the Krasnoyarsk region, an expansion of the tick area to the north was reported [28]. The increasing incidence of TBE was also observed in Poland [31], Norway [32], Sweden [33], Finland [34], and Mongolia [35]. In Central Europe [36], and in Italy [37], TBEV area was expanding to areas with higher elevation above sea level, which was also attributed to the warming climate. Recently, human cases of TBE were registered in Western Europe [38,39]. In October 2019, TBEV in ticks in the UK was reported for the first time [40]. In the summer of 2019, a new TBEV focus in Denmark was identified [41].

Controlling the spread of TBEV requires an understanding of the factors that have affected its spread and evolution in the past. Previous studies of TBEV evolution suggested different, sometimes conflicting hypotheses, each with apparently robust statistical support. The number of available TBEV sequences has more than doubled over the last 6 years. This expanded dataset allows a more detailed and informative analysis of the evolution of the TBEV.

## 2. Materials and Methods

**Figure 1:**
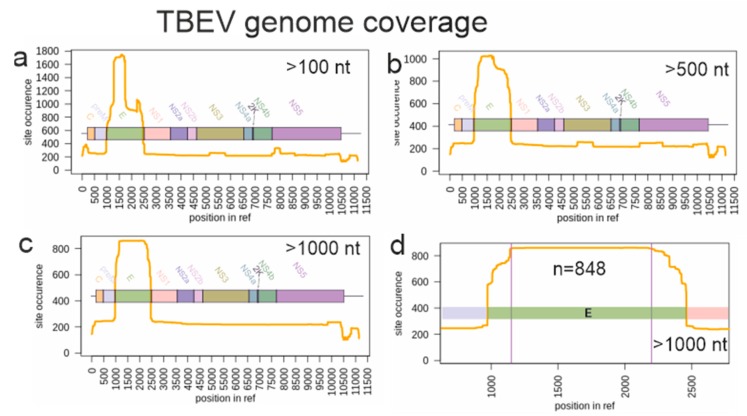
Genome layout and TBEV genome fragments represented in GenBank as of June 2019. Y-axis indicates the sequence coverage (the number of known sequences) for each genome position shown in X-axis. (**a**) Sequences shorter than 100 nucleotides were omitted from the analysis. (**b**) Sequences shorter than 500 nucleotides were omitted. (**c**) Sequences shorter than 1000 nucleotides were omitted. (**d**) The genomic region selected for the phylogenetic analysis is limited by two vertical lines.

Prior to analysis, sequences represented in GenBank as of June 2019 and longer than 1000 nucleotides and aligning with genome positions 1150–2200 in the reference sequence NC_001672 were selected (*n* = 848) (Figure 1c,d). Datasets for TBEV genome coverage visualization were generated using open-source tools (Supplementary File S1). For further analysis, sequences with known dates of sample collection (*n* = 683) that were automatically annotated from the GenBank records were used. Then, 233 sequences with dates known from publications or personal communications were manually added to this dataset. Identical sequences from the same geographical region were removed. The final dataset consisted of 551 sequences, each 1028 nucleotides long.

There were no significant traces of the recombination according to RDP4 software [42]. Maximum likelihood (ML) phylogenetic inference was performed using IQ-TREE [43]. The best-fit model was automatically chosen using ModelFinder [44] implemented in IQ-TREE package (v.1.6.1) according to the Bayesian information criterion. Ultrafast bootstrap (BB) approximation (1000 replicates) was chosen to assess statistical robustness for internal branching order in the phylogeny [45]. Clades with support more than 95% were proposed to be reliable.

All trees were rooted using the best-fitting-root according to the residual mean squared function in Tempest (v1.5) software [46]. The presence of a temporal signal was assessed using TempEst v1.5 software using the residual mean squared best-fitting-root function.

Bayesian model averaging was performed using the bModelTest package [47]. Next, different clock assumptions (relaxed log-normal, relaxed exponential, and strict) and population models (constant size, exponential growth, and Bayesian skyline) were compared by a Bayes factors test. Marginal likelihood was calculated using Path Sampler implemented in the Beast 2.5.1 [48]. The combinations of models with the highest Bayes factor were strict clock/Bayesian skyline, relaxed log-normal/exponential growth, and relaxed log-normal/constant size for European, Siberian, and Far-Eastern subtypes, respectively.

The length of MCMC chains varied from 100,000,000 to 500,000,000 million generations to reach the convergence of parameters. These estimates were checked using Tracer (v1.5) and indicated by an effective sample size >200. Tree visualization and annotation was performed using FigTree v1.4.2.

Past population dynamics was estimated in BEAST 1.10 using the Skygrid coalescent model [49]. Bayesian Skygrids were visualized using R scripts.

Table 1 was generated using information about geographically distant but genetically close viruses from the Bayesian phylogenetic trees (Figures 5–7). Identity values were measured for selected viruses using the BlastN algorithm [50]. Direct air distance between places of virus collection was calculated from geographical coordinates using https://www.distancecalculator.net/. Most recent common ancestor times (tMRCA) for selected pairs of viruses were extracted from the first common node in the appropriate Bayesian phylogenetic trees.

## 3. Results

### 3.1. Sample Selection and Its Effect on the Ancestry Inference Estimates

Phylogenetic studies on TBEV are traditionally done using the sequence encoding for the envelope glycoprotein E (1488 nt), and many studies use only partial E region sequence. As a result, coverage of different TBEV genomic regions in GenBank is very uneven (Figure 1). There are just about 200 full-genome sequences and about 1750 sequences of the E region. Preliminary tests indicated that the phylogenetic resolution was not significantly compromised when using complete or near-complete E region compared to full genome sequences. On the other hand, the number of sequences (and, thus, the geographic coverage) was much higher for E region. A 1000 nt long fragment of E genome region represented by about 800 sequences (Figure 1d) was arbitrarily selected as a balance between dataset size (number of sequences) and sequence length (phylogenetic resolution).

The number of known TBEV sequences has increased significantly over the last 10 years. In 2010, there were just 38 complete ORF sequences and 202 complete E protein coding sequences in GenBank. This number rose to 215 complete ORF sequences and 464 complete E protein sequences in 2019. Small datasets can introduce a significant bias into phylogenetic inference [51]. The increased number of TBEV sequences allowed evaluating the potential effect of sample bias on the early TBEV phylogenetic reconstructions by analysis of simulated reduced datasets.

**Figure 2:**
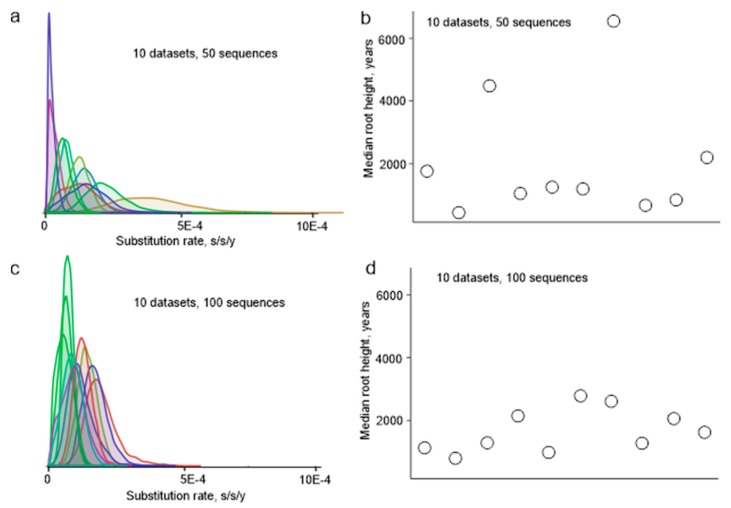
(**a**) Substitution rates among 10 random datasets (50 viruses each) of partial E protein sequences (1028 nt). (**b**) Root height in 10 random datasets (50 viruses each) of partial E protein sequences (1028 nt). (**c**) Substitution rates among 10 random datasets (100 viruses each) of partial E protein sequences (1028 nt). (**d**) Root height in 10 random datasets (100 viruses each) of partial E protein sequences (1028 nt).

Random sampling of sequences from a large dataset was not a valid approach to simulate the actual sample bias in sequence datasets, because some research groups deposited dozens of almost identical sequences to GenBank, while others reported just a few unique sequences, and random sampling would preferentially pick sequences from large studies. To simulate the sample bias that could have existed 10 years ago more realistically, we designed an algorithm that mimics a situation when only few of the actual virus sequencing studies were done. Sequences from one study are usually deposited to GenBank at once and have sequential accession numbers. Blocks of sequences with sequential accession numbers were sampled from all available non-identical E protein coding sequences fragments with known isolation dates (551 sequences of 1028 nt) until there were at least 50 sequences in a simulated dataset. Ten random datasets were created. Then, these simulated datasets were used to infer phylogenetic relations of TBEV. The estimated mean substitution rate varied among simulated datasets more than 15-fold, between 0.22 × 10^−4^ and 3.84 × 10^−4^ substitutions per site per year (s/s/y), sometimes with non-overlapping 95% high probability density (HPD) intervals (Figure 2a). Similarly, the height of the trees (age of the most recent common ancestor (tMRCA) of TBEV) differed significantly (Figure 2b). Therefore, sample bias has a profound effect on the evolutionary estimates, which is not fully reflected by formal statistical support within an analysis. It is noteworthy that when the simulated datasets included 100 instead of 50 sequences, the effect of bias was much less pronounced (Figure 2c,d), and the inferred substitution rates varied just 3.6-fold, between 0.54 × 10^−4^ and 1.96 × 10^−4^. It is not possible to estimate the actual bias in the currently available dataset of 551 sequences, but the simulation results suggest that it is significantly smaller than in the dataset available just a decade ago.

### 3.2. Suitability of TBEV Genomic Data for Molecular Clock Analysis

Bayesian phylogenetic analysis uses the molecular clock model, which is the statistical description of the relationship between observed genetic distances and time [46]. In the absence of this relationship in the dataset, temporal estimates may be misleading.

**Figure 3:**
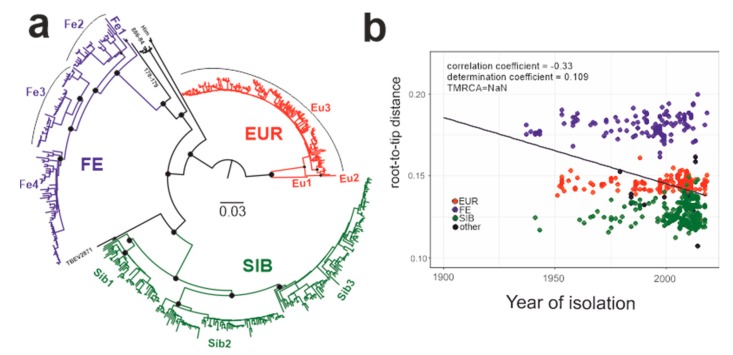
(**a**) Maximum likelihood tree for TBEV species (1028 nt). Black circles indicate high-level nodes that were supported by UFBoot values over 95% [45]. (**b**) Association between root-to-tip distance and time of isolation for the whole TBEV species.

**Figure 4:**
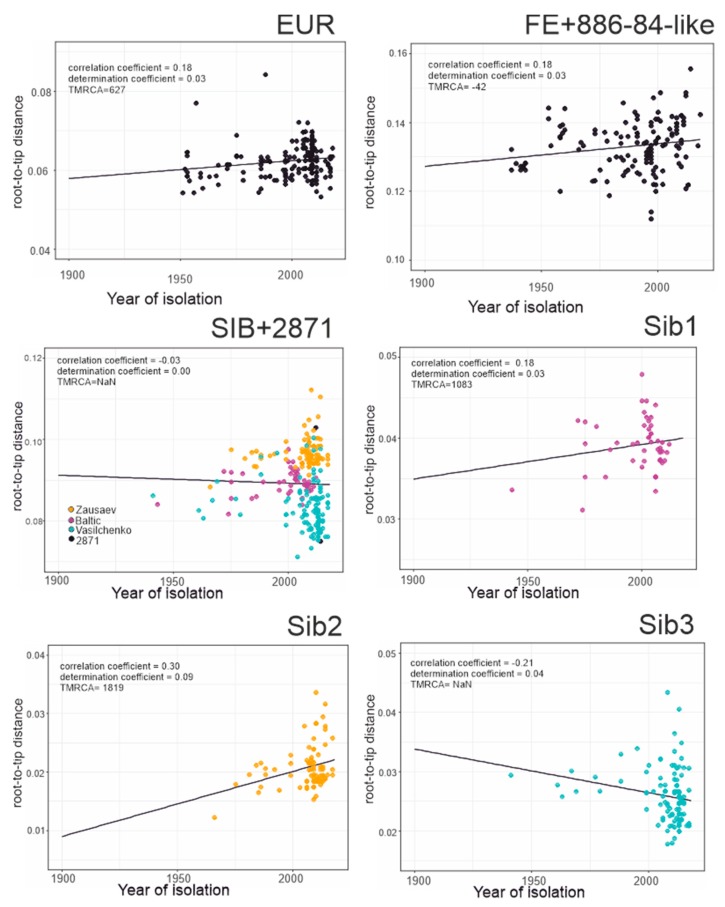
Association between root-to-tip distance and time of isolation for separated TBEV groups. EUR–European subtype; FE + 886-84-like–Far-Eastern subtype and 886-84 and 178-179 strains; SIB + 2871–Siberian and TBEV-2871 strain; Sib1, Sib2, and Sib3—separated lineages of Siberian subtype.

This study analyzed 551 near-complete E-gene sequences of TBEV (Figure 3a), including 181 sequences of the European subtype (Figure 3a, red branches, Figure 3b red circles), 232 sequences of the Siberian subtype (Figure 3a, green branches, Figure 3b green circles), and 125 sequences of the Far-Eastern subtype (Figure 3a, blue branches, Figure 3b blue circles) as well as 13 sequences with an uncertain subtype assignment (Figure 3a, black branches, Figure 3b black circles). The association between root-to-tip distance and time of isolation demonstrated the absence of a temporal signal for the TBEV species as a whole (Figure 3b), precluding molecular clock analysis of all TBEV subtypes in one dataset. Thus, the TBEV species had to be divided into three datasets: European subtype, Far-Eastern subtype (including “uncertain” Baikalian strains 886-84 and 178-179), and Siberian subtype (including the “uncertain” sequence TBEV-2871). The European and Far-Eastern subtypes demonstrated a weak temporal signal (Figure 4) and could be analyzed using the molecular clock. For the whole Siberian subtype, genetic distances were independent of isolation time, similar to the entire TBEV, but two of the three Siberian subtype groups (Figure 3a, Sib1 and Sib2) had a temporal structure (Figure 4). For consistency with the subtype assignment, the Siberian subtype was analyzed as a whole, but dating of the deep node was regarded as unreliable.

### 3.3. Phylogenetic Analysis

The geographic distribution of sequences corresponded to that generally known for the subtypes [21]. The topology of the overall maximum likelihood phylogenetic tree (Figure 3a) corresponded to earlier studies [12,19,25,52]. The Siberian subtype was grouped with the Far-Eastern subtype. As said above, the TBEV species had to be divided into three datasets prior to Bayesian phylogenetic analysis: European subtype, Far-Eastern subtype (including “uncertain” Baikalian strains 886-84 and 178-179) and Siberian subtype (including the “uncertain” sequence TBEV-2871). The mean nucleotide substitution rates inferred using individual subtype datasets were 0.891 × 10^−4^, 1.452 × 10^−4^, and 1.449 × 10^−4^ s/s/y for the European, Siberian, and Far-Eastern subtypes, respectively. The rates for the Sib1 (Baltic lineage) and Sib2 (prototype strain Zausaev) subsets were 1.25 × 10^−4^ (0.09 × 10^−4^–2.57 × 10^−4^) and 2.06 × 10^−4^ (0.43 × 10^−4^–3.57 × 10^−4^), respectively. These rates were in line with previous studies [52,53].

Root heights, or the most recent common ancestors of individual subtypes (tMRCAs), were 1632 (814–4790) 722 (401–1272) and 888 (510–1395) years ago for the European, Siberian, and Far-Eastern subtypes, respectively. Previously Siberian and Far-Eastern subtypes tMRCAs were suggested to exist 809 and 778 years ago, respectively [53]. Our results are in concordance with these estimates. It should be noted that European subtype tMRCA existence time has not been estimated previously upon finding the new genetically distant virus in the Netherlands in 2017 (GenBank #LC171402) [54].

**Figure 5:**
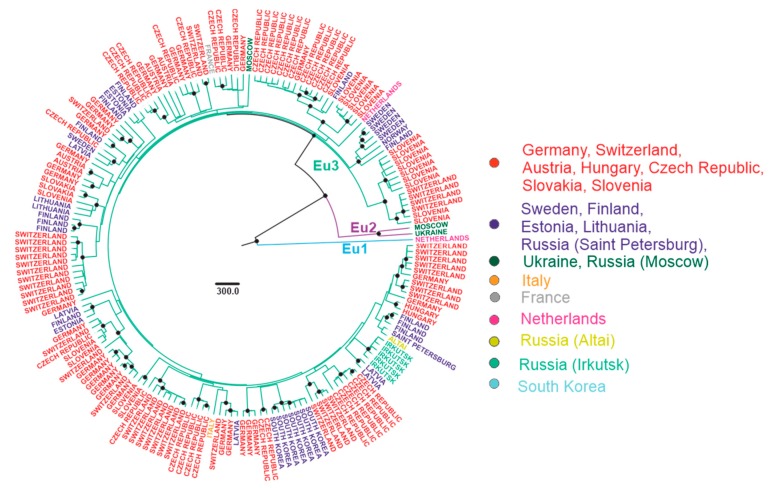
Bayesian phylogenetic analysis of near-complete E-gene sequences of European TBEV. Branches are color-shaded according to described groups. Tree tips are named according to the region of isolation. Countries or country regions of virus sampling were grouped into nine color-coded geographical regions. Node posterior probabilities above 95% are shown by black circles at the relevant nodes.

**Figure 6:**
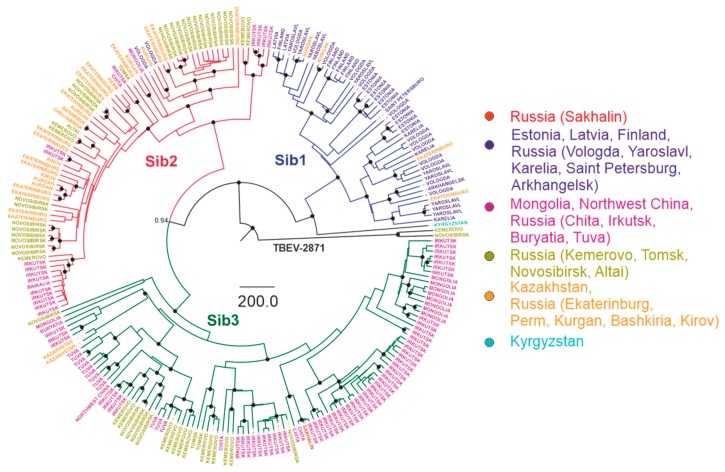
Bayesian phylogenetic analysis of near-complete E-gene sequences of Siberian TBEV. Branches are color-shaded according to described groups. Tree tips are named according to region of isolation. Countries or country regions of virus sampling were grouped into six color-coded geographical regions. Node posterior probabilities above 95% are shown by black circles at the relevant nodes.

**Figure 7:**
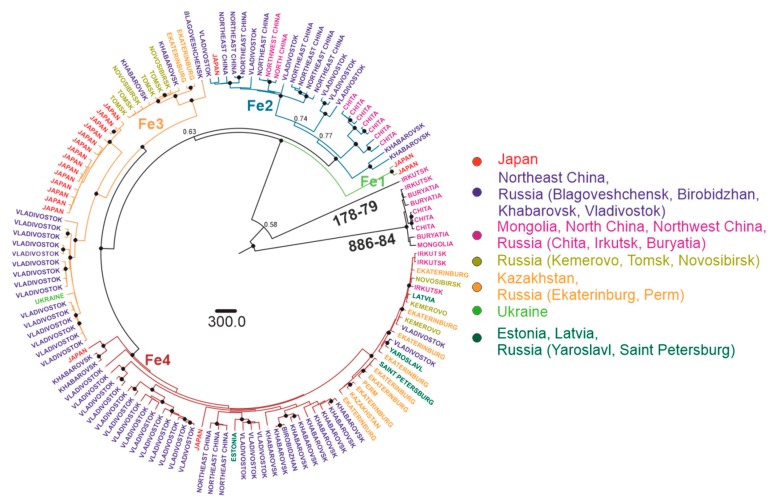
Bayesian phylogenetic analysis of near-complete E-gene sequences of Far-Eastern TBEV. Branches are color-shaded according to described groups. Tree tips are named according to region of isolation. Countries or country regions of virus sampling were grouped into seven color-coded geographical regions. Node posterior probabilities above 95% are shown by black circles at the relevant nodes.

The most commonly used taxonomic term for dividing TBEV species into lower taxonomic levels is a “subtype” [6,13,21,55,56,57]. Clusters with significant statistical support were observed within the subtype, further termed as “subtype groups” or “subgroups”. A strict phylogenetic distance criterion for TBEV sub-subtype, or subgroup, classification has not been defined yet. To facilitate further discussion, viruses within subtypes were divided into a few major subgroups of comparable age of corresponding tree nodes with reliable statistical support. Three such subgroups were found within the European subtype (Eu1–3), three within Siberian (Sib1–3), and four within Far-Eastern (Fe1–4) subtype (Figure 5, Figure 6 and Figure 7). Viruses within these subgroups had their tMRCA around 386–654 years ago. Therefore, phylogenetic grouping, rather than a single distance criterion, can be suggested for sub-subtype classification of TBEV. Many of the tree nodes within these subgroups had low statistical support; therefore, lower level classification requires more profound and detailed investigation.

In order to facilitate tree analysis, isolates were color-coded according to isolation regions, using arbitrary areas about 1000–1500 km across for each color (Figure 5, Figure 6 and Figure 7). Countries with territory more than 5,000,000 sq km, Russia (296 viruses) and China (16 viruses), were subdivided into smaller regions. Total TBEV area was thus divided into 15 non-overlapping areas (right panels in Figure 5, Figure 6 and Figure 7).

**Figure 8:**
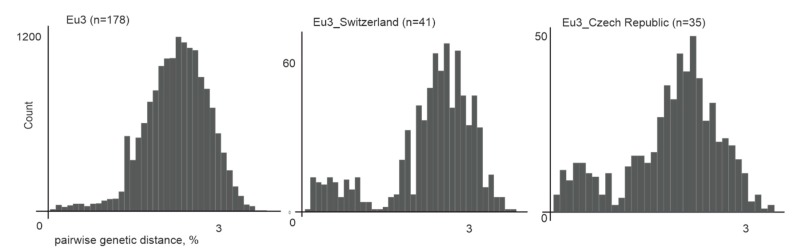
Pairwise genetic distances for all Eu3 representatives (*n* = 178), Eu3 from Switzerland (*n* = 41), and Eu3 from the Czech Republic (*n* = 35).

All European TBEV were divided into three subgroups, further termed Eu1–3 (Figure 5). Eu1 was represented by a sole divergent sequence from the Netherlands. Eu2 had only two sequences from Russia and Ukraine, as more sequences from that subgroup were removed by identity filtration (see above). Most isolates were within Eu3, which could not be divided into any further subgroups due to poor statistical support of many nodes within that group (Figure 5). This could be explained by low genetic diversity of isolates, 178 sequences had on average 98% genetic identity. On the other hand, a relatively short length of E genome region was used for analysis. There was no geographic pattern within Eu3, and some of the genetically closest viruses within that group were separated by thousands of kilometers (Table 1). Moreover, the diversity of viruses within a small country, such as Switzerland or the Czech Republic, was comparable to the diversity of viruses of this group found all over Eurasia (Figure 8). The tMRCA for Eu3 existed 419 (233–1204) years ago.

For the Siberian subtype (Figure 6), there were three major groups: Sib1, or the Northwestern group (Northwestern Russia, Baltic countries), with the tMRCA 412 (231–742) years ago; Sib2 (prototype strain Zausaev), or the Ural-Siberian group, with the tMRCA 386 (209–693) years ago; and Sib3 (prototype strain Vasilchenko), or the Siberia-Mongolian group, with the tMRCA 521 (288–925) years ago. Within each of these phylogenetic groups, there were highly similar viruses isolated at distant locations that had very recent common ancestors (Figure 6, Table 1). Most inter-regional transfers (common nodes from distant areas) within this group were dated between 11 and 83 years back.

For the Far-Eastern subtype, there were four subgroups predominantly found in a specific area: the Japan subgroup (Fe1, tMRCA 64 (31–120) years ago), Manchuria (Northeast China and Russian Far East) region subgroup (Fe2, tMRCA 528 (387–1043) years ago), Pacific-Siberia subgroup (Fe3, tMRCA 528 (317–843) years ago and Pacific-Ural subgroup (Fe4, tMRCA 479 (281–743) years ago). Similar to the Siberian type, within these groups, there were single isolates from very distant locations, such as Vladivostok-Crimea, Vladivostok-Estonia, Khabarovsk-Novosibirsk, etc. (Figure 7, Table 1).

### 3.4. Population Dynamics

The phylogenetic analysis found multiple long-distance transfers and suggested thorough mixing, especially within the European subgroup Eu3. To further explore the evolution of the TBEV over time, past population dynamics were inferred for the three major TBEV subtypes using the Bayesian Skygrid model, a non-parametric coalescent model that estimates the effective population size over time [49]. Noteworthy, it is not clear how much the observed viral diversity corresponds to the general sample of all existing TBEV, and the results described below were obtained by analyzing sequences represented in GenBank as of June 2019.

**Figure 9:**
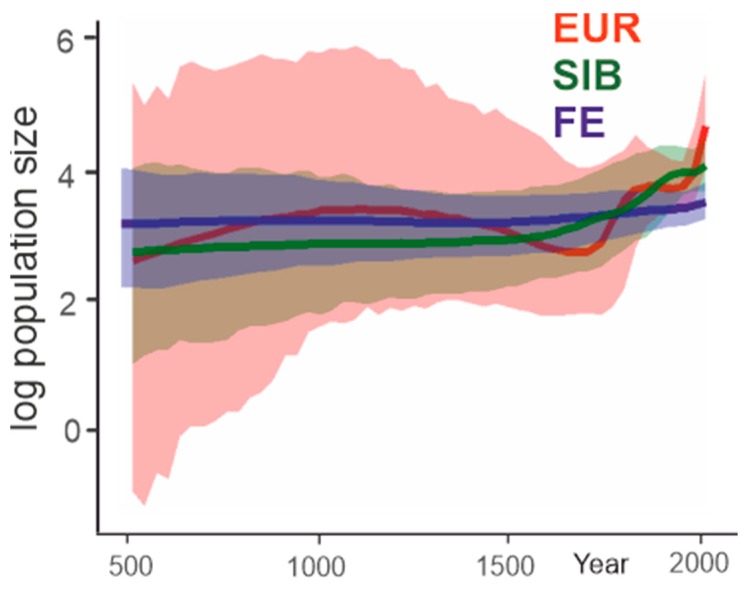
Past population dynamics inferred for the three major TBEV subtypes using the Bayesian Skygrid model.

The European subtype demonstrated explosive growth of population size in two periods. From 1711 to 1834, the virus population size increased about eight-fold (Figure 9). Then, until about the mid-20th century, the population size was stable. The second explosive growth period started in 1956, and the population size increased further about eight-fold. The inferred Far-Eastern subtype population size has slightly increased by around 20% over the last 50 years. The Siberian subtype population size has increased about four-fold during the last 200 years. Therefore, an increase of virus population was suggested independently for all three TBEV subtypes and was most pronounced in the European subtype, which is also most thoroughly intermixed geographically. A high number of recent tree nodes (Figure 5, Figure 6 and Figure 7) corresponded to a rapid recent increase in the population size of the European and, to a lesser extent, Siberian subtype inferred by a Bayesian Skygrid analysis.

## 4. Discussion

Analysis of the sample bias effect on phylogenetic inference indicates that the current dataset can give much more reliable evolutionary estimates than was possible just 10–15 years ago. Nevertheless, within all TBEV subtypes, there were “lone” viruses that branched close to the root and were represented by one or few isolates. This, together with recent findings of divergent Baikal group viruses and a novel subtype in China, implies that our knowledge of TBEV diversity is far from exhaustive, and further findings may question the current understanding of the spread of ancient TBEV and its diversification.

The contemporary sample of known TBEV sequences gives a novel view of the diversity and spread of the known types. Although virus transfers and findings of virus subtypes far away from their “name regions” have been described previously, it is now clear that long-distance transfers are a systematic pattern rather than anecdotal events. This is best exemplified by the virus diversity observed in Switzerland or the Czech Republic. These two countries yielded most of the known Eu3 subgroup sequences, and the diversity of viruses found within each of these countries is comparable to that of the whole Eu3 subgroup, which is prevalent all over Central and Eastern Europe (Figure 8). It is very likely that more extensive sampling would show comparably high virus diversity in other regions of Europe as well. This observation suggests frequent and systematic virus trafficking. In the Siberian and Far-Eastern subtypes, there were examples of both “cosmopolitan” subgroups with evidence of multiple transfers all over Northern Eurasia, and “endemic” clusters, represented by dozens of diversified sequences, yet confined to a single region.

Phylogenetic analysis allowed identifying subgroups based on a well-supported grouping at a comparable tree depth (ca. 300–500 years) in all three TBEV subtypes. This grouping is less useful for the European subtype, because almost all sequences belong to the Eu3 subgroup. However, in the Siberian and Far-Eastern subtypes, this allowed dividing viruses into several comparably divergent and prevalent groups, most of them with their “typical” sub-regions. It is interesting that the tMRCA of these major subtypes was similar, and relatively recent, in all three subtypes.

Most of the deep tree nodes within all three established TBEV subtypes dated less than 300 years back at the intra-subgroup level. This could be explained by either population expansion-extinction cycles, which has regularly “reset” the existing virus diversity, by saturation of synonymous mutations, or indeed by recent emergence of most of the known TBEV diversity. The first hypothesis could be supported by long tree branches leading to the main subtypes (Figure 3a) and by the global fluctuations in human infection incidence observed for TBEV. Such global-scale bottlenecks are typical to, for example, picornaviruses [58] or rabies virus [59]. On the other hand, it is hard to imagine how all diversity of a zoonotic virus that features small endemic foci (due to the nature of natural hosts and vectors) could shrink to a few variants on a continental scale. Mutation saturation, which can indeed compromise dating of other viruses, is also unlikely here, because the substitution rate in TBEV is about 10^−4^ s/s/y. In other words, about 1% of substitutions can be accumulated in about 100 years, and the sequence diversity within subtypes does not exceed 10%. Therefore, this factor could affect long term evolutionary estimates (dating over 2000 years back), but not timing of more recent events. Thus, recent dating of most of the tree nodes might indeed correspond to the recent emergence of most of the known TBEV diversity.

Within all subtypes and within most of the smaller groups in these subtypes, there were examples of long- and medium-range virus transfers, which occurred within the last 300 years, and some of them within the last few decades (Table 1). Here, we define long- and medium-range virus transfers as the detection of genetically close viruses in geographically non-overlapping regions (right panel, Figure 5, Figure 6 and Figure 7). Dating of these events may lack precision due to both calculation uncertainty and TBEV biology (e.g., a possibility of long-term virus preservation during a tick diapause), but their very recent occurrence is evident. It is noteworthy that more examples of such long-distance transfers have been reported previously, but were not accounted for in this study due to the short length of analyzed sequences. For example, TBEV-Fe was found in ticks collected in the Republic of Moldova [15], and all three TBEV subtypes were found in Crimea [16]. Such transfers could be mediated by human activity or by naturally migrating animals, including bats and birds, which were infected or carried infected ticks. It was demonstrated that TBEV in ticks can be transported by birds [60]. In that study, 57 of 91 fieldfares captured in the south of Western Siberia were carrying a total of 510 ticks, and more than half of them contained TBEV RNA. Also, a recent detection of TBEV in Great Britain was attributed to migratory birds that hypothetically transferred this virus from Norway [40]. Thus, birds cannot be discarded as a TBEV dissemination vector. However, this mechanism should have produced some sort of mixing gradient among regions, with more common medium-range than long-range transfers. There are many extremely long-distance transfers without any intermediate virus isolations, for example between Europe and South Korea or between Central Siberia and Sakhalin Island in the Pacific (Table 1). Such long-distance “quantum” transfers are not compatible with a mixing gradient and with natural transfers. Human-assisted transfer is a more plausible explanation in this case. It could be mediated, for example, by trafficking of domestic animals carrying infected ticks. Another more recent example of anthropogenic activity that could facilitate TBEV spread comes from mid-20th century Soviet Union. It has been suggested that a large-scale introduction of novel wildlife species to new habitats to improve hunting capacity has complemented to the intermixing of TBEV subtypes [61]. Very limited geographic clustering within the Eu3 subgroup as compared to other subgroups would then be compatible with higher population density and intensity of human activity in Europe.

A hypothesis of anthropogenic dissemination of TBEV evident from phylogenetic clustering analysis is also supported by population dynamics analysis (Figure 9). On the other hand, isolated findings of rare divergent TBEV variants may suggest that the known TBEV diversity is largely represented by the “sinantropic” lineages, which were indeed spread via human activity, while TBEV in (poorly explored) sylvatic foci is not as dependent on human activity as could be inferred from the sequences that are predominant in GenBank.

Phylogenetic analysis suggests that virus transfers were the key mechanism shaping spatial diversity of TBEV. If such a model has existed for a long time, it should have resulted in a thoroughly intermixed Eurasian TBEV population, where identification of the types would not have been possible, but this is not the case. This observation is compatible with a recent diversification of TBEV in general and with the current rapid European TBEV expansion to novel areas and habitats [20,39,40,62,63,64,65]. Predicted past population dynamics (Figure 9) is also in concordance with this data. Thus, the European TBEV population is undergoing a roughly exponential growth phase now. From a practical point of view, this implies that the established areas of TBEV subtypes (and, therefore, the disease epidemiology) would be increasingly eroded in the 21st century. This process might be less extreme in Siberia and the Far East, but the evolutionary events, such as recent diversification, long-distance transfers, and inferred population growth, are generally paralleled in all three major subtypes.

Rapid population dynamics of TBEV amplifies other ecological and epidemiological risks. There is a risk that novel types, such as the recently found “Chinese” variant [18], can spread via human activity and emerge as novel healthcare threats. Additionally, TBEV may be introduced to novel habitats and acquire new vectors. Many tick species can potentially transmit TBEV [66,67,68], and it is not known if their limited role in supporting endemic TBEV is due to ecological conditions (e.g., absence of a complete niche with vectors and hosts) or because the virus has not yet been introduced to new niches.

## 5. Conclusions

Recently, a significant expansion of TBEV distribution range has been recorded. These local emergence incidents are reflected by findings of generally highly dynamic TBEV evolution in space (long-distance transfers and mixing within an area) and time (very recent diversification and population expansion). These findings are not expected, especially in the case of TBEV, which has been long considered an ancient (at least Ice Age-old) zoonosis strongly bound to very specific ecological niches. Results of bioinformatics analysis presented here, together with multiple field findings, suggest that TBEV may be regarded as an emerging disease.

## Figures and Tables

**Table 1:** Closely related viruses found at distant locations.

Strain	Nearest Neighbor in Terms of Genetic Distance (Nearly Identical Sequences from the Same Place Were Omitted)	Nt Sequence Identity between Strain of Interest and Its Nearest Neighbor, %	Approximate Direct Air Distance, km	tMRCA (95% HPD)
**EUR**				
Eu3 MH663428 Russia Moscow 2017	Eu3 JQ654701 Slovenia 1992	98.1	2000	1681 (1228–1799)
Eu3 MK801803 Finland 2005	Eu3 JF501414 Czech Republic 1986	99,3	1300	1876 (1541–1937)
Eu3 AF091007 France Alsace 1975	Eu3 JF501408 Czech Republic 1978	99,4	500	1898 (1745–1950)
Eu3 AJ319583 Latvia 1997	Eu3 MH704574 Germany Battaune 2017	99,91	950	1967 (1882–1996)
Eu3 KY069126 Russia Altai 1986	Eu3 AF091005 Russia Saint Petersburg 1951	99.91	3200	1934 (1851–1951)
Eu3 MH021184 Netherlands 2016	Eu3 AF091005 Russia Saint Petersburg 1951	98.39	1750	1624 (814–1793)
Eu3 KJ994330 Italy 2013	Eu3 AF091005 Russia Saint Petersburg 1951	99.43	1900	1724 (1468–1836)
**SIB**				
Sib3 KF826916 Russia Sakhalin 2011	Sib3 KC422663 Russia Chita 2000	100.00	2200	1993 (1973–2000)
Sib1 FJ214123 Russia Ekaterinburg 2006	Sib1 FJ214145 Russia Yaroslavl 2001	98.54	1200	1855 (1699–1940)
Sib1 GQ845418 Russia Ekaterinburg 2009	Sib1 Baltic GQ845439 Russia Yaroslavl 2008	98.73	1200	1883 (1764–1057)
Sib1 FJ214131 Russia Kurgan 2007	Sib1 FJ214153 Russia Vologda 2007	99.90	1600	1995 (1956–2007)
Sib1 KY319395 Russia Kurgan 2010	Sib1 GQ845440 Russia Yaroslavl 2008	100.00	1600	2003 (1986–2008)
Sib2 GQ845427 Russia Ekaterinburg 2009	Sib2 MG598843 Russia Novosibirsk 2013	98.86	1400	1873 (1751–1944)
Sib2 FJ214150 Russia Kurgan 2007	Sib2 FJ214137 Russia Vologda 1975	98.10	1600	1882 (1766–1956)
Sib2 AF527415 Russia Tomsk Zausaev 1985	Sib2 KR633032 Russia Kirov 2012	98.54	2100	1757 (1560–1875)
Sib2 JF274481 Mongolia Bulgan 2010	Sib2 MG598825 Russia Novosibirsk 2011	98.01	1700	1756 (1543–1879)
Sib2 KC417475 Russia Irkutsk 2010	Sib2 KR633015 Russia Kemerovo 2014	98.39	1200	1872 (1741–1952)
Sib2 MF161158 Russia Irkutsk 2015	Sib2 GQ845421 Russia Ekaterinburg 2009	99.05	2800	1863 (1741–1933)
Sib3 KF826916 Russia Sakhalin 2011	Sib3 KC422663 Russia Chita 2000	100.00	2200	1993 (1972–2000)
**FE**				
Fe2 LC440460 Japan-Nanporo 2018	Fe2 GU121642 Russia Vladivostok 2008	98.96	800	1946 (1884–1989)
Fe3 JX987281 Russia Khabarovsk 1973	Fe3 KJ739731 Russia Tomsk Sorex araneus 2006	100.00	3400	1949 (1916–1969)
Fe3 FJ214120 Russia Ekaterinburg 1959	Fe3 KJ739731 Russia Tomsk Sorex araneus 2006	99.43	1500	1898 (1831–1944)
Fe3 AF091008 Ukraine 1987	Fe3 AY169390 Russia Prymorye Prymorye-332 human blood 1991	99.53	7300	1905 (1850–1951)
Fe4 LC440459 Japan- Sapporo Ixodes ovatus 2017	Fe4 KP869172 Russia-Khabarovsk 1985	97.91	780	1810 (1639–1927)
Fe4 DQ393779 Estonia Laanemaa 1996	Fe4 AB049345 Russia-Vladivostok 1999	98.48	6900	1835 (1725–1919)
Fe4 FJ214119 Russia Ekaterinburg 1943	Fe4 FJ214119 Russia- SaintPetersburg 1943	99.81	1800	1924 (1901–1940)
Fe4 FJ214147 Russia Yaroslavl 1989	Fe4 AF091013 Russia Vladivostok 1979	99.72	6200	1954 (1917–1977)
Fe4 AF091016 Latvia 1977	Fe4 FJ214133 Russia Kemerovo 1967	99.72	3700	1961 (1948–1967)

## Supplementary Materials

The supplementary materials are available online at https://www.mdpi.com/1999-4915/12/2/247/s1.

## References

[1] Beauté J., Spiteri G., Warns-Petit E., Zeller H. (2018). Tick-borne encephalitis in Europe, 2012 to 2016. Eurosurveillance.

[2] Federal Service for Surveillance on Consumer Rights Protection and Human Wellbeing. www.rospotrebnadzor.ru/activities/statistical-materials/.

[3] Lindquist L., Vapalahti O. (2008). Tick-borne encephalitis. Lancet.

[4] Kriz B., Hubalek Z., Marek M., Daniel M., Strakova P., Betasova L. (2015). Results of the Screening of Tick-Borne Encephalitis Virus Antibodies in Human Sera from Eight Districts Collected Two Decades Apart. Vector Bome Zoonotic Dis..

[5] Maikova G.B., Chernokhaeva L.L., Rogova Y.V., Kozlovskaya L.I., Kholodilov I.S., Romanenko V.V., Esyunina M.S., Ankudinova A.A., Kilyachina A.S., Vorovitch M.F. (2019). Ability of inactivated vaccines based on far-eastern tick-borne encephalitis virus strains to induce humoral immune response in originally seropositive and seronegative recipients. J. Med. Virol..

[6] Ecker M., Allison S.L., Meixner T., Heinz F.X. (1999). Sequence analysis and genetic classification of tick-borne encephalitis viruses from Europe and Asia. J. Gen. Virol..

[7] Charrel R.N., Attoui H., Butenko A.M., Clegg J.C., Deubel V., Frolova T.V., Gould E.A. (2004). Tick-borne virus diseases of human interest in Europe. Clin. Microbiol. Infect..

[8] Yoshii K., Song J.Y., Park S.-B.B., Yang J., Schmitt H.-J.J. (2017). Tick-borne encephalitis in Japan, Republic of Korea and China. Emergy Microbes Infect..

[9] Jaaskelainen A.E., Castren J., Subbotina N., Sironen T., Alekseev A.N., Vapalahti O., Murueva G.B., Vaheri A., Alitalo I. (2010). Tick-borne encephalitis virus in ticks in Finland, Russian Karelia and Buryatia. J. Gen. Virol..

[10] Golovljova I., Vene S., Sjölander K.B., Vasilenko V., Plyusnin A., Lundkvist Å. (2004). Characterization of tick-borne encephalitis virus from Estonia. J. Med. Virol..

[11] Pukhovskaya N., Morozova O., Belozerova N., Bakhmetyeva S., Vysochina N., Zdanovskaya N., Ivanov L. (2017). Comparative analysis of genomes of tick-borne encephalitis virus strains isolated from mosquitoes and ticks. Vopr. Virusol..

[12] Tkachev S.E., Babkin I.V., Chicherina G.S., Kozlova I.V., Verkhozina M.M., Demina T.V., Lisak O.V., Doroshchenko E.K., Dzhioev Y.P., Suntsova O.V. (2019). Ticks and Tick-borne Diseases Genetic diversity and geographical distribution of the Siberian subtype of the tick-borne encephalitis virus. Ticks Tick Bome Dis..

[13] Muto M., Bazartseren B., Tsevel B., Dashzevge E., Yoshii K., Kariwa H. (2015). Isolation and characterization of tick-borne encephalitis virus from Ixodes persulcatus in Mongolia in 2012. Ticks Tick Bome Dis..

[14] Briggs B.J., Atkinson B., Czechowski D.M., Larsen P.A., Meeks H.N., Carrera J.P., Duplechin R.M., Hewson R., Junushov A.T., Gavrilova O.N. (2011). Tick-Borne Encephalitis Virus, Kyrgyzstan. Emerg. Infect. Dis..

[15] Ponomareva E.P., Mikryukova T.P., Gori A.V., Kartashov M.Y., Protopopova E.V., Chausov E.V., Konovalova S.N., Tupota N.L., Gheorghita S.D., Burlacu V.I. (2015). Detection of Far-Eastern subtype of tick-borne encephalitis viral RNA in ticks collected in the Republic of Moldova. J. Vector Bome Dis..

[16] Yurchenko O.O., Dubina D.O., Vynograd N.O., Gonzalez J.-P. (2017). Partial Characterization of Tick-Borne Encephalitis Virus Isolates from Ticks of Southern Ukraine. Vector Bome Zoonotic Dis..

[17] Demina T., Dzhioev Y., Kozlova I., Verkhozina M., Tkachev S., Doroshchenko E., Lisak O., Paramonov A., Zlobin V. (2012). genotypes 4 and 5 of the tick-Borne encephalitis Virus: Features of the genome Structure and Possible Scenario for its formation. Vopr. Virusol..

[18] Dai X., Shang G., Lu S., Yang J., Xu J. (2018). A new subtype of eastern tick-borne encephalitis virus discovered in Qinghai-Tibet Plateau, China. Emerg. Microbes Infect..

[19] Adelshin R.V., Sidorova E.A., Bondaryuk A.N., Trukhina A.G., Sherbakov D.Y., White R.A., Andaev E.I., Balakhonov S.V. (2019). “886-84-like” tick-borne encephalitis virus strains: Intraspecific status elucidated by comparative genomics. Ticks Tick Bome Dis..

[20] Dekker M., Laverman G.D., de Vries A., Reimerink J., Geeraedts F. (2019). Emergence of tick-borne encephalitis (TBE) in the Netherlands. Ticks Tick Bome Dis..

[21] Ruzek D., Avšič Županc T., Borde J., Chrdle A., Eyer L., Karganova G., Kholodilov I., Knap N., Kozlovskaya L., Matveev A. (2019). Tick-borne encephalitis in Europe and Russia: Review of pathogenesis, clinical features, therapy, and vaccines. Antivir. Res..

[22] Imhoff M., Hagedorn P., Schulze Y., Hellenbrand W., Pfeffer M., Niedrig M. (2015). Review: Sentinels of tick-borne encephalitis risk. Ticks Tick Bome Dis..

[23] Pukhovskaya N.M., Morozova O.V., Vysochina N.P., Belozerova N.B., Bakhmetyeva S.V., Zdanovskaya N.I., Seligman S.J., Ivanov L.I. (2018). Tick-borne encephalitis virus in arthropod vectors in the Far East of Russia. Ticks Tick Bome Dis..

[24] Kholodilov I., Belova O., Burenkova L., Korotkov Y., Romanova L., Morozova L., Kudriavtsev V., Gmyl L., Belyaletdinova I., Chumakov A. (2019). Ixodid ticks and tick-borne encephalitis virus prevalence in the South Asian part of Russia (Republic of Tuva). Ticks Tick Bome Dis..

[25] Heinze D.M., Gould E.A., Forrester N.L. (2012). Revisiting the Clinal Concept of Evolution and Dispersal for the Tick-Borne Flaviviruses by Using Phylogenetic and Biogeographic Analyses. J. Virol..

[26] Chernokhaeva L.L., Kholodilov I.S., Pakskina N.D. (2016). Current distribution area of tick-borne encephalitis in the russian federation. Med. Virol..

[27] Suss J. (2008). Tick-borne encephalitis in Europe and beyond--the epidemiological situation as of 2007. Euro Surveill..

[28] Revich B., Tokarevich N., Parkinson A.J. (2012). Climate change and zoonotic infections in the Russian Arctic. Int. J. Circumpolar Health.

[29] Tokarevich N.K., Tronin A.A., Blinova O.V., Buzinov R.V., Boltenkov V.P., Yurasova E.D., Nurse J. (2011). The impact of climate change on the expansion of Ixodes persulcatus habitat and the incidence of tick-borne encephalitis in the north of European Russia. Glob. Health Action.

[30] Bugmyrin S.V., Bespyatova L.A., Korotkov Y.S., Burenkova L.A., Belova O.A., Romanova L., Kozlovskaya L.I., Karganova G.G., Ieshko E.P. (2013). Distribution of Ixodes ricinus and I. persulcatus ticks in southern Karelia (Russia). Ticks Tick Borne Dis..

[31] Grzybek M., Alsarraf M., Tołkacz K., Behnke-Borowczyk J., Biernat B., Stańczak J., Strachecka A., Guz L., Szczepaniak K., Paleolog J. (2018). Seroprevalence of TBEV in bank voles from Poland—A long-term approach. Emerg. Microbes Infect..

[32] Sidorenko M., Radzievskaja J., Rosef O., Paulauskas A. (2018). Investigation of the tick-borne encephalitis virus in Norway. Biologija.

[33] Jaenson T.G.T., Hjertqvist M., Bergström T., Lundkvist A. (2012). Why is tick-borne encephalitis increasing? A review of the key factors causing the increasing incidence of human TBE in Sweden. Parasit. Vectors.

[34] Tonteri E., Jokelainen P., Matala J., Pusenius J., Vapalahti O. (2016). Serological evidence of tick-borne encephalitis virus infection in moose and deer in Finland: Sentinels for virus circulation. Parasit. Vectors.

[35] Baasandavga U., Badrakh B., Burged N., Davaajav O., Khurelsukh T., Barnes A., Ulaankhuu U., Nyamdorj T. (2019). A case series of fatal meningoencephalitis in Mongolia: Epidemiological and molecular characteristics of tick-borne encephalitis virus. West. Pacific Surveill Response J..

[36] Brabec M., Daniel M., Malý M., Danielová V., Kříž B., Kott I., Beneš Č. (2017). Analysis of meteorological effects on the incidence of tick-borne encephalitis in the Czech Republic over a thirty-year period. Virol. Res. Rev..

[37] Martello E., Mannelli A., Ragagli C., Ambrogi C., Selmi M., Ceballos L.A., Tomassone L. (2014). Range expansion of Ixodes ricinus to higher altitude, and co-infestation of small rodents with Dermacentor marginatus in the Northern Apennines, Italy. Ticks Tick Bome Dis..

[38] de Graaf J.A., Reimerink J.H., Voorn G.P., Bij de Vaate E.A., de Vries A., Rockx B., Schuitemaker A., Hira V. (2016). First human case of tick-borne encephalitis virus infection acquired in The Netherlands, July 2016. Euro Surveill.

[39] Velay A., Solis M., Kack-Kack W., Gantner P., Maquart M., Martinot M., Augereau O., De Briel D., Kieffer P., Lohmann C. (2018). A new hot spot for tick-borne encephalitis (TBE): A marked increase of TBE cases in France in 2016. Ticks Tick Bome Dis..

[40] Holding M., Dowall S.D., Medlock J.M., Carter D.P., Pullan S.T., Lewis J., Vipond R., Rocchi M.S., Baylis M., Hewson R. (2020). Tick-Borne Encephalitis Virus, United Kingdom. Emerg. Infect. Dis..

[41] Agergaard C.N., Rosenstierne M.W., Bødker R., Rasmussen M., Andersen P.H.S., Fomsgaard A. (2019). New tick-borne encephalitis virus hot spot in Northern Zealand, Denmark, October 2019. Euro Surveill..

[42] Martin D.P., Murrell B., Golden M., Khoosal A., Muhire B. (2015). RDP4: Detection and analysis of recombination patterns in virus genomes. Virus Evol..

[43] Nguyen L.-T., Schmidt H.A., von Haeseler A., Minh B.Q. (2015). IQ-TREE: A Fast and Effective Stochastic Algorithm for Estimating Maximum-Likelihood Phylogenies. Mol. Biol. Evol..

[44] Kalyaanamoorthy S., Minh B.Q., Wong T.K.F., von Haeseler A., Jermiin L.S. (2017). ModelFinder: Fast model selection for accurate phylogenetic estimates. Nat. Methods.

[45] Minh B.Q., Nguyen M.A.T., von Haeseler A. (2013). Ultrafast Approximation for Phylogenetic Bootstrap. Mol. Biol. Evol..

[46] Rambaut A., Lam T.T., Max Carvalho L., Pybus O.G. (2016). Exploring the temporal structure of heterochronous sequences using TempEst (formerly Path-O-Gen). Virus Evol..

[47] Bouckaert R.R., Drummond A.J. (2017). bModelTest: Bayesian phylogenetic site model averaging and model comparison. BMC Evol. Biol..

[48] Bouckaert R., Heled J., Kuhnert D., Vaughan T., Wu C.H., Xie D., Suchard M.A., Rambaut A., Drummond A.J. (2014). BEAST 2: A Software Platform for Bayesian Evolutionary Analysis. PLoS Comput. Biol..

[49] Hill V., Baele G. (2019). Bayesian Estimation of Past Population Dynamics in BEAST 1.10 Using the Skygrid Coalescent Model. Mol. Biol. Evol..

[50] Johnson M., Zaretskaya I., Raytselis Y., Merezhuk Y., McGinnis S., Madden T.L. (2008). NCBI BLAST: A better web interface. Nucleic Acids Res..

[51] Vakulenko Y., Deviatkin A., Lukashev A. (2019). The Effect of Sample Bias and Experimental Artefacts on the Statistical Phylogenetic Analysis of Picornaviruses. Viruses.

[52] Subbotina E.L., Loktev V.B. (2012). Molecular evolution of the tick-borne encephalitis and Powassan viruses. Mol. Biol..

[53] Tkachev S.E., Chicherina G.S., Golovljova I., Belokopytova P.S., Tikunov A.Y., Zadora O.V., Glupov V.V., Tikunova N.V. (2017). New genetic lineage within the Siberian subtype of tick-borne encephalitis virus found in Western Siberia, Russia. Infect. Genet. Evol..

[54] Jahfari S., De Vries A., Rijks J.M., Van Gucht S., Vennema H., Sprong H., Rockx B. (2017). Tick-borne encephalitis virus in ticks and roe deer, the Netherlands. Emerg. Infect. Dis..

[55] Gritsun T.S., Lashkevich V.A., Gould E.A. (2003). Tick-borne encephalitis. Antiviral Res..

[56] Leonova G.N., Belikov S.I., Kondratov I.G., Takashima I. (2013). Comprehensive assessment of the genetics and virulence of tick-borne encephalitis virus strains isolated from patients with inapparent and clinical forms of the infection in the Russian Far East. Virology.

[57] Smura T., Tonteri E., Jääskeläinen A., Von Troil G., Kuivanen S., Huitu O., Kareinen L., Uusitalo J., Uusitalo R., Hannila-Handelberg T. (2019). Recent establishment of tick-borne encephalitis foci with distinct viral lineages in the Helsinki area, Finland. Emerg. Microbes Infect..

[58] Lukashev A.N., Vakulenko Y.A. (2017). Molecular evolution of types in non-polio enteroviruses. J. Gen. Virol..

[59] Deviatkin A.A., Lukashev A.N., Poleshchuk E.M., Dedkov V.G., Tkachev S.E., Sidorov G.N., Karganova G.G., Galkina I.V., Shchelkanov M.Y., Shipulin G.A. (2017). The phylodynamics of the rabies virus in the Russian Federation. PLoS ONE.

[60] Mikryukova T.P., Moskvitina N.S., Kononova Y.V., Korobitsyn I.G., Kartashov M.Y., Tyuten’kov O.Y., Protopopova E.V., Romanenko V.N., Chausov E.V., Gashkov S.I. (2014). Surveillance of tick-borne encephalitis virus in wild birds and ticks in Tomsk city and its suburbs (Western Siberia). Ticks Tick. Bome Dis..

[61] Kovalev S.Y., Chernykh D.N., Kokorev V.S., Snitkovskaya T.E., Romanenko V.V. (2009). Origin and distribution of tick-borne encephalitis virus strains of the Siberian subtype in the Middle Urals, the north-west of Russia and the Baltic countries. J. Gen. Virol.

[62] Brockmann S., Oehme R., Buckenmaier T., Beer M., Jeffery-Smith A., Spannenkrebs M., Haag-Milz S., Wagner-Wiening C., Schlegel C., Fritz J. (2018). A cluster of two human cases of tick-borne encephalitis (TBE) transmitted by unpasteurised goat milk and cheese in Germany, May 2016. Eurosurveillance.

[63] Kuivanen S., Smura T., Rantanen K., Kämppi L., Kantonen J., Kero M., Jääskeläinen A., Jääskeläinen A.J., Sane J., Myllykangas L. (2018). Fatal Tick-Borne Encephalitis Virus Infections Caused by Siberian and European Subtypes, Finland, 2015. Emerg. Infect. Dis..

[64] Jääskeläinen A., Tonteri E., Pieninkeroinen I., Sironen T., Voutilainen L., Kuusi M., Vaheri A., Vapalahti O. (2016). Siberian subtype tick-borne encephalitis virus in Ixodes ricinus in a newly emerged focus, Finland. Ticks Tick Borne Dis..

[65] Makenov M., Karan L., Shashina N., Akhmetshina M., Zhurenkova O., Kholodilov I., Karganova G., Smirnova N., Grigoreva Y., Yankovskaya Y. (2019). First detection of tick-borne encephalitis virus in Ixodes ricinus ticks and their rodent hosts in Moscow, Russia. Ticks Tick Borne Dis..

[66] Hoogstraal H. (1966). Ticks in relation to human diseases caused by viruses. Annu. Rev. Entomol..

[67] Takeda T., Ito T., Chiba M., Takahashi K., Niioka T., Takashima I. (1998). Isolation of Tick-Borne Encephalitis Virus from Ixodes ovatus (Acari: Ixodidae) in Japan. J. Med. Entomol..

[68] Yun S.M., Song B.G., Choi W., Park W.I., Kim S.Y., Roh J.Y., Ryou J., Ju Y.R., Park C., Shin E.H. (2012). Prevalence of tick-borne encephalitis virus in ixodid ticks collected from the republic of Korea during 2011–2012. Osong Public Health Res. Perspect..

