# Supplementary material for: Tick-Borne Encephalitis Virus: An Emerging Ancient Zoonosis?
Source: Viruses. 2020 Feb 23;12(2):247. doi: 10.3390/v12020247 (PMC7077300; doi:10.3390/v12020247)
Supplement: Supplementary file 1 [file viruses-12-00247-s001.pdf]

```
makeblastdb -in ~/work/tbev/NC_001672.fas -dbtype nucl -title NC_001672 -
out ~/soft/blast+/db/NC_001672; cd ~/soft/blast+/db ; blastn -max_hsps 5 -
max_target_seqs 1 -word_size 7 -query ~/work/tbev/txid11084_200619.fas -db
NC_001672 -evalue 0.0000000001 -out
~/work/tbev/blastn_txid11084_200619_vs_NC_001672_tmp -outfmt '6 qseqid
sseqid pident length qlen slen evalue qstart qend sstart send' ; cd ~/work/tbev
; cut -f 1,10,11 blastn_txid11084_200619_vs_NC_001672_tmp >
tbev_coord_in_NC_001672 ; cut -f 2,3 tbev_coord_in_NC_001672 > tmp; sed -
i 's/\t/-/g' tmp ; perl -pe 's/(\d+)-(\d+)/join(",",$1..$2)/eg' tmp > tmp1 ; sed -i
':a;N;$!ba;s/\n/,/g' tmp1 ; sed -i 's/,/\n/g' tmp1 ; sort -g tmp1 > tmp2; sed -i
'/^$/d' tmp2; uniq -c tmp2 > tmp3 ; sed -i 's/^ *//g' tmp3; awk '{print $2,$1}'
tmp3 > GB_occurence_per_NC_001672_sites.txt ;
```
